# Restarting pre-exposure prophylaxis (PrEP) for HIV: a systematic review and meta-analysis

**DOI:** 10.1016/j.eclinm.2024.102647

**Published:** 2024-05-17

**Authors:** Reuben Kiggundu, Qi Rui Soh, Warittha Tieosapjaroen, Christopher K. Fairley, Joseph D. Tucker, Weiming Tang, Lei Zhang, Jason J. Ong

**Affiliations:** a Melbourne Sexual Health Centre, Alfred Health, Melbourne, Australia; b School of Translational Medicine, Faculty of Medicine, Nursing and Health Sciences, Monash University, Melbourne, Australia; c University of Melbourne, Melbourne, Victoria, Australia; d Faculty of Infectious and Tropical Diseases, London School of Hygiene and Tropical Medicine, London, United Kingdom; e Institute for Global Health and Infectious Diseases, University of North Carolina at Chapel Hill, Chapel Hill, NC, USA; f Clinical Medical Research Center, Children's Hospital of Nanjing Medical University, Nanjing, Jiangsu Province, China

**Keywords:** HIV, Prevention, PrEP, Pre-exposure prophylaxis, Restarting

## Abstract

**Background:**

High coverage of pre-exposure prophylaxis (PrEP) will reduce HIV transmission and help end the HIV/AIDS pandemic. However, PrEP users face challenges, including long-term adherence. The study aimed to document the proportions of individuals who restart HIV PrEP after they stop and the reasons for restarting PrEP.

**Methods:**

This study is a systematic review and meta-analysis. We systematically searched CINAHL, Embase, Emcare, Global Health, Medline, Scopus, and PsychINFO for peer-reviewed with no date restrictions. A grey literature search was conducted through Google search, a search of abstract books of AIDS conferences and the websites of WHO and UNAIDS. The data search was conducted in April 2023 and updated in February 2024. Two authors extracted data on the proportion of people who stopped and then restarted PrEP, reasons for restarting, and strategies to support people restarting PrEP. Two authors appraised the data using the Joanna Briggs Institute Appraisal Tools. We used a random-effects meta-analysis to pool estimates of restarting. We conducted meta-regression to determine potential sources of heterogeneity. This study is registered with PROSPERO, CRD42023416777. However, we deviated from our original plan as we did not identify enough studies for strategies to support restarting PrEP (primary objective). Subsequently, we revised our plan to strengthen our secondary objective to quantify the proportion of people who stopped and restarted PrEP, and explore possible reasons for its heterogeneity.

**Findings:**

Of 988 studies, 30 unique studieswere included: 27 reported the proportion restarting PrEP, and of these, 7 also reported reasons for restarting PrEP, and 3 studies reported only on the reasons for restarting PrEP. No study evaluated interventions for restarting PrEP. For the meta-analysis, we included 27 studies. Most studies were from high-income countries (17/27, 63%) or the USA (15/27, 56%). Overall, 23.8% (95% CI: 15.9–32.7, *I*^*2*^ = 99.8%, N = 85,683) of people who stopped PrEP restarted PrEP. There was a lower proportion of restarting in studies from middle-income countries compared to high-income countries (adjusted odds ratio (aOR) 0.6, 95% CI: 0.50–0.73, p < 0.001). There was higher restarting in studies from Africa compared to the USA (aOR 1.55, 95% CI: 1.30–1.86), heterosexual populations compared to men who have sex with men or transgender women (aOR 1.50, 95% CI: 1.25–1.81, p < 0.001) and in studies defining restarting as those who had stopped PrEP for >1 month compared to those who stopped <1 month (aOR 1.20, 95% CI: 1.06–1.36, p < 0.001). Reasons for restarting PrEP included perceived higher risk for HIV acquisition and removal of barriers to access PrEP. In terms of quality assessment, overall, both randomised controlled trials had a low risk of bias, while the observational studies used in the meta-analysis had some potential risk of bias related to not explicitly addressing potential confounders (15/25, 60%) or not describing strategies to address incomplete follow-up (24/25, 96%).

**Interpretation:**

About a quarter of people who stopped PrEP would restart, with substantial variation across countries and populations. It is important to understand the motivations and contextual factors influencing restarting PrEP and the support systems to enable restarting PrEP for those at ongoing risk.

**Funding:**

Australian National Health and 10.13039/501100000265Medical Research Council.

**Research in context**

**Evidence before this study**

Prexposure prophylaxis (PrEP) is a highly effective and safe method to prevent HIV. Current literature reports significant proportions of people who stop using PrEP. However, it is unclear if those who stopped PrEP would restart PrEP. We searched PubMed in April 2023 using the terms “HIV’, “AIDS”, “pre-exposure prophylaxis”, “stop”, “restart”, “resuming” and “reinitiate” to identify reviews for restarting PrEP. One systematic review published in 2022 reported a pooled proportion of restarting PrEP of 37%. However, this review only included eight studies and did not assess factors associated with restarting PrEP or the reasons why individuals chose to restart PrEP.

**Added value of this study**

We identified 30 relevant studies: 27 reported the proportion restarting PrEP, of these, 7 also reported reasons for restarting PrEP, 3 studies reported only on the reasons for restarting PrEP. Overall, 23.8% (95% CI: 15.9–32.7, *I*^*2*^ = 99.8%, N = 85,683) of people who stopped PrEP restarted PrEP. The observed heterogeneity was mostly explained by the location of the study, population type and the definition of restarting. The main reasons individuals restarted PrEP included a perceived increase in individual risk for HIV acquisition (e.g., with new sexual partners or sexual partners returning from travel) and the removal of structural barriers to access PrEP. No studies documented interventions to support people in restarting PrEP.

**Implications of all the available evidence**

There remains a paucity of evidence to understand how individuals who stop PrEP choose to restart PrEP or not. Our review uncovered that a quarter of people who stopped PrEP would restart, which has implications for PrEP programs to ensure those who remain at risk for HIV can be supported to restart PrEP in a timely manner.

## Introduction

HIV is a leading cause of morbidity and mortality globally.^1^ The World Health Organization estimates that between 32.9 and 51.3 million people have died from AIDS-related illnesses since the start of the epidemic in 1981, with an estimated 630,000 AIDS-related deaths in 2022. Currently, over 39.0 million people are living with HIV. Despite efforts to achieve global targets towards epidemic control, over 1.3 million [1 million–1.7 million] new infections were reported in 2022.^1^ Strengthening HIV prevention strategies,^2^ is critical to achieving the Joint United Nations Programme on HIV/AIDS (UNAIDS) 95-95-95 goals of awareness of HIV status, being on treatment and achieving viral suppression.^3^

Improving access to antiretrovirals for people with HIV and scaling up pre-exposure prophylaxis (PrEP) are some of the key HIV prevention strategies to eliminate HIV transmission. PrEP, when used properly, is highly effective in preventing new HIV infections.^4^ Various models of PrEP delivery are under implementation, including daily PrEP, on-demand PrEP, and the recent authorisation of long-acting PrEP.^4^^,^^5^ Despite an increase of 182% in the use of PrEP since 2018, gaps in its availability and use remain, with only 28% of the target of 3 million in low- and middle-income countries receiving PrEP in 2022.^1^ Furthermore, to derive the maximal benefit from PrEP, users must adhere (i.e., take their PrEP correctly) and persist (i.e., take PrEP for the required duration) during periods of risk for HIV acquisition.

Adherence to and continuation of PrEP can be challenging, with studies reporting adherence to PrEP of 50%–80%^6^^,^^7^ and the proportion of PrEP discontinuation at six months was reported as 37% in a systematic review done in 2018.^8^ This is comparable to the 2022 systematic review reporting 41% PrEP discontinuation within six months after starting it.^9^ Geographic and socioeconomic variations in findings have been documented, with stigma, dosing challenges, side effects, and inaccurate risk perception as drivers of PrEP non-adherence.^10^ Given that PrEP adherence is essential for the high efficacy of PrEP, it is vital that individuals are supported to restart PrEP, especially whenever there is ongoing or a recurrence of risk for HIV acquisition.^10^ None of the previous systematic reviews examined correlates for restarting PrEP nor reasons for restarting. Further understanding of patterns of PrEP use can help inform PrEP programs to support users to restart PrEP when it is stopped inappropriately given ongoing risk for HIV or whenever there is a recurrence of risk for HIV acquisition in the future. Thus, our study aimed to provide a pooled proportion of users who restarted PrEP, document reasons for restarting PrEP, and identify strategies to support restarting PrEP.

## Methods

This systematic review has been registered at the International Prospective Register of Systematic Reviews (PROSPERO ID: CRD42023416777). The review was done according to the Cochrane guidance on conducting systematic reviews.^11^ This review is reported per Preferred Reporting Items for Systematic Reviews and Meta-Analyses (PRISMA) guidelines.^12^

### Search methods

Ovid MEDLINE, Ovid EMBASE, Scopus, Ovid Emcare, Web of Science, Ebsco CINAHL, and Global Health were searched between 6th April 2013 and 13th April 2023 and updated in February 2024. The grey literature search was done through Google search and search of abstract books AIDS conferences and websites of WHO and UNAIDS. The search terminology revolved around four key aspects: ‘HIV’, “PrEP”, “restarting” and ‘reinitiation’. Appendix 1 shows the complete search strategy. The inclusion criteria were any study that contained primary data on the proportion of people who restarted PrEP, reasons for restarting PrEP, or described interventions for restarting PrEP. We excluded studies that did not have primary data (e.g., opinion papers, review papers) and studies with less than 10 participants.

Titles and abstracts were independently assessed via Covidence for eligibility by two reviewers (QS, RK). Another reviewer (JO) resolved any discrepancies. We did not identify any studies about interventions for re-starting PrEP and focused the study outcomes on the proportions of individuals re-starting and reasons for re-starting. The outcomes related to interventions for restarting PrEP are not reported in this manuscript due to a lack of data.

### Data extraction

A data extraction file was created in Microsoft Excel, and the following information was collected: country of study, setting of the study, study population, duration of stopping before PrEP restarting, definitions of PrEP restarting, reasons for restarting, the proportion of people restarting and specific interventions for PrEP restarting. We did not define a specific duration for how long someone had to have stopped PrEP before restarting but directly extracted each study's definition (as depending on the setting, people may be using PrEP on-demand or periodically for defined periods of risks). Data extraction was conducted by two reviewers (QS, RK), and another reviewer (JO) resolved any discrepancies.

### Meta-analysis methods

We used descriptive statistics to summarise the characteristics of the studies included. We used random effects meta-analysis to calculate the pooled proportion of individuals restarting PrEP. Inter-study heterogeneity was assessed using the *I*^*2*^ statistic. We conducted meta-regression to explore reasons for heterogeneity. Study bias was assessed using a funnel plot and Egger's test. STATA (StataCorp. 2021. Stata Statistical Software: Release 17. College Station, TX: StataCorp LLC) was used to conduct all statistical analyses. The STATA command utilised for meta-analysis was ‘metaprop’.

### Qualitative synthesis

Regarding data for reasons for restarting PrEP, we used inductive thematic analysis to summarise the main themes from the studies. The extracted data was grouped into common codes followed by themes. RK designed the code and coded the data, QS reviewed the codes and JO resolved discrepancies. The themes were then used for report writing.

### Quality assessment of included studies

Two reviewers (QS and RK) assessed each study's quality using the relevant critical appraisal tool from the Joanna Briggs Institute.^13^ Both randomised controlled trials were assessed using the tool entitled “Randomised Controlled Trials”, while the remaining studies were assessed using the tool entitled “Cohort Studies”.

### Role of the funding source

The funders did not have any role in the study design, collection, analysis, interpretation of the data, writing the report or decision to submit the paper for publication.

## Results

### Results of search

**Fig. 1:**
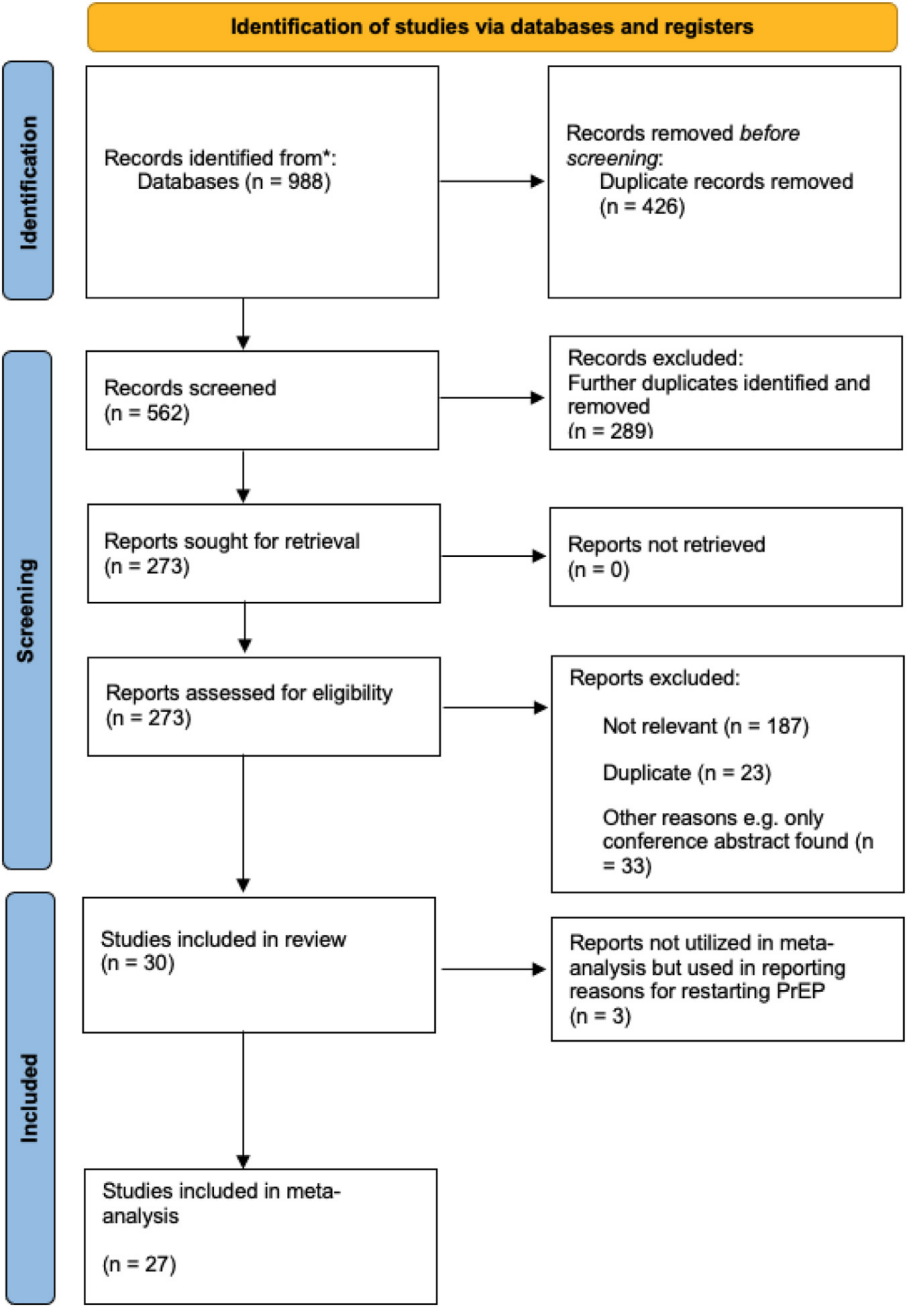
**PRISMA flowchart for meta-analysis**.

The search identified 988 studies, and we included 30 unique studies: 27 reported the proportion restarting PrEP, and of these, 7 also reported reasons for restarting PrEP, and 3 studies reported only on the reasons for restarting PrEP (Fig. 1).

### Characteristics of included studies

Table 1 summarises the main characteristics of the studies included in the meta-analysis. For studies used in our meta-analysis, most were from high-income countries (17/27, 63%), the United States of America (USA) (15/27, 56%), and almost all were observational studies (25/27, 93%). Further details of the included studies are provided in Table 2.

**Table 1:** Characteristics of the included studies.

Characteristic	Studies included in the meta-analysis of restarting PrEP (N = 27)		Studies with reasons for restarting PrEP (N = 10)^b^	
	n	%	n	%
**Country income level**^f^				
High	17	63.0	6	60
Middle	8	29.6	4	40
Mixed (Low and Middle-income countries^a^)	2	7.4	0	0
**Region of the world**				
Africa	10	37.0	4	40
Americas^c^	15	55.5	6	60
Europe	1	3.7	0	0
Western Pacific	1	3.7	0	0
**Study setting**				
Community	4	14.8	6	60
Online	1	3.7		
Clinic	19	70.3	4	40
Combined	3	11.1		
**Study type**				
Randomised clinical trials^d^	2	7.4	1	10
Non-experimental (Cross-sectional and cohort studies)	25	92.6	9	90
**Study population**				
Heterosexual women	1	3.7		
AGYW	4	14.8	1	10
MSM/TGW	8	29.6	4	40
Mixed^e^	14	51.8	5	50

AGYW, Adolescent Girls and Young Women; MSM, men who have sex with men; TGW, Transgender Women; PrEP, pre-exposure prophylaxis.

a Multisite studies completed in a combination of low- and middle-income countries, e.g., Uganda and Kenya.

b Overlap of seven studies with systematic review.

c All studies in the Americas region were from USA.

d Only data from PrEP users were extracted.

e Mixed population with MSM, Bisexual, transgender, heterosexual men and women.

f Per the World Bank Classification.^14^

**Table 2:** Further details of studies included.

Studies included in the meta-analysis (N = 27)							
Author (Year)	Country income level	Region of the world	Study setting	Study type	Study population	Definition of restarting PrEP	Duration of observation of study participants
Celum, 2020^15^	Middle	African	Community	RCT	AGYW	Missed refill due to missed visit, or ≥21 days not taking PrEP as documented in discontinuation form. Women who missed a refill but had a subsequent visit where they accepted PrEP were considered to have restarted PrEP.	12 months
Celum, 2022^16^	Middle	African	Online	Cohort	AGYW	Taking PrEP again after a gap of 15 days	6 months
Chakare, 2019^17^	Middle	African	Community	Cross-sectional	AGYW	Not specified	Not specified
Chase, 2023^18^	High	Americas	Clinic	Cohort	Mixed	Starting PrEP after being off for more than 30 days	28 months
Coyer, 2020^19^	High	European	Clinic	Cohort	MSM and Transgender	Not specified	9 months 28 days/∼10 months
Dombrowski, 2017^20^	High	Americas	Clinic	Cohort	MSM	Not specified	26 months
Glidden, 2016^21^	High	Americas	Community	Cohort	MSM	Not specified	18 months
Hall, 2022^22^	High	Americas	Community	Cohort	Gay, Bisexual	PrEP re-initiation was identified as a visit where a participant reported currently taking PrEP after having previously indicated discontinuation. Visits were ∼6 months apart	61 months
Heffron, 2016^23^	Mix (Low and Middle)	African	Clinic	Cohort	Heterosexual	Not specified	2 years/∼ 24 months
Hevey, 2018^24^	High	Americas	Clinic	Cohort	Mixed but predominantly MSM	Not specified	67 months
Hojila, 2021^25^	High	Americas	Clinic	Cohort	Mixed but predominantly MSM	Not specified	80 months
Irungu, 2021^26^	Middle	African	Clinic	RCT	Heterosexual	Participants who had failed to attend the previous quarterly yearly refill visit	34 months
Jin, 2021^27^	High	Western Pacific	Clinic	Cohort	Mixed but predominantly MSM	Not specified	26 months
Johnson, 2022^28^	High	Americas	Clinic	Cross-sectional	Mixed but predominantly MSM	Not specified	Not specified
Koester, 2018^29^	High	Americas	Clinic	Cohort	Heterosexual	Not specified	Not specified
Koss, 2020^30^	Mix (Low and Middle)	African	Community	Cohort	Heterosexual	Restarted by week 72	72 weeks/∼16.6 Months
Krakower, 2019^31^	High	Americas	Clinic	Cohort	Mixed but predominantly MSM	Not specified	59 months
Liu, 2016^32^	High	Americas	Community	Cohort	MSM	Not specified	48 weeks/∼11.0466 months
Marcus, 2016^33^	High	Americas	Clinic	Cohort	Mixed but predominantly MSM	Not specified	35 months
Rao, 2023^34^	Middle	African	Clinic	Cohort	AGYW, FSW	Not specified	60 months
Reed, 2021^35^	Middle	African	Clinic	Cohort	Mixed	Not specified	3 years/∼36 months
Rousseau, 2021^36^	Middle	African	Clinic	Cohort	AGYW	Participants who initiated PrEP and pharmacy records show a break in PrEP use for more than 30 days before a PrEP pill pick-up at a later clinic visit	4 years/∼ 48 months (study only specifies 2017–2020)
Serota, 2019^37^	High	Americas	Clinic	Cross-sectional	MSM	PrEP discontinuation was defined as ≥14 days off of PrEP after initiation	24 months
Shover, 2020^38^	High	Americas	Clinic	Cohort	MSM	Not specified	4 months
Unger, 2022^39^	High	Americas	Clinic	Cohort	Mixed but predominantly MSM	Not specified	∼10 months
Wahome, 2022^40^	Middle	African	Community	Cohort	TGW	Not specified	24 months
Whitefield, 2018^41^	High	Americas	Clinic	Cohort	Gay and Bi-sexual men	Not specified	24 months

**Studies with reasons for restarting (N** = **10)**							
Author (Year)	Country income level	Region of the world	Study setting	Study type	Study population	Definition of restarting PrEP	Duration of observation of study participants
Celum, 2020^15^	Middle	Africa	Community	Cohort	AGYW	Missed refill due to missed visit, or ≥21 days not taking PrEP as documented in discontinuation form. Women who missed a refill but had a subsequent visit where they accepted PrEP were considered to have restarted PrEP	12 months
Corneli, 2022^42^	Middle	Africa	Community	Cohort	Cis-gender women	Not specified	
Gaspar, 2022^43^	High	Americas	Community	Cohort	Gay and Bi-sexual men	Not specified	
Hall, 2022^22^	High	Americas	Community	Cohort	Gay, Bisexual	PrEP re-initiation was identified as a visit where a participant reported currently taking PrEP after having previously indicated discontinuation. Visits were ∼6 months apart	61 months
Rousseau, 2021^36^	Middle	Africa	Clinic	Cohort	AGYW	Participants who initiated PrEP and pharmacy records show a break in PrEP use for more than 30 days before a PrEP pill pick-up at a later clinic visit	4 years/∼ 48 months (study only specifies 2017–2020)
Rowe, 2022^44^	High	Americas	Clinic	Cross-sectional	Heterosexual	Not specified	
Shover, 2020^38^	High	Americas	Clinic	Cross-sectional	MSM	Not specified	4 months
Unger, 2022^39^	High	Americas	Clinic	Cross-sectional	Mixed but predominantly MSM	Not specified	∼10 months
Wahome, 2022^40^	Middle	Africa	Community	Cohort	TGW	Not specified	24 months
Whitefield, 2018^41^	High	Americas	Clinic	Cohort	Gay and Bi-sexual men	Not specified	24 months

AGYW, adolescent girls and young women; MSM, men who have sex with men; RCT, randomized clinical trial.

### Proportion of restarting

**Fig. 2:**
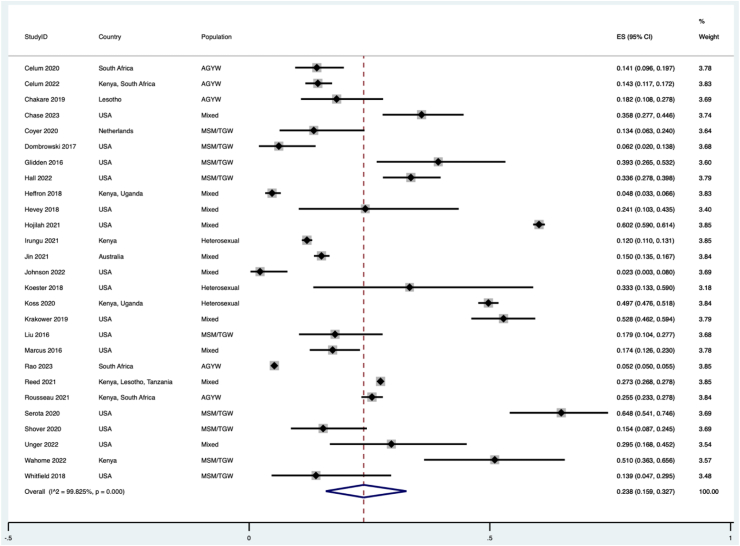
**Forest plot of the proportion of people who restarted pre-exposure prophylaxis (N = 27)**. AGYW, adolescent girls and young women; MSM, men who have sex with men; TGW, transgender women; USA, United States of America.

Fig. 2 summarises the estimated proportion of people who restarted PrEP by country and population. Overall, 23.8% (95% CI: 15.9–32.7, *I*^*2*^ = 99.8%, N = 85,683) of people who stopped PrEP restarted PrEP. Supplementary Figure S1 demonstrates there was no evidence of publication bias (Egger's test, p = 0.51). To explore the causes of heterogeneity, Table 3 presents the univariable and multivariable meta-regression results. This revealed that there was a lower proportion of restarting in studies from middle-income countries (compared to high-income countries) and a higher restarting from studies from Africa (compared to the USA), heterosexual populations (compared to MSM/TGW), and in studies which defined restarting as those who had stopped PrEP for more than a month (compared to those who stopped up to one month). We present the sub-group analyses for country income level (Supplementary Figure S2), world region (Supplementary Figure S3), population type (Supplementary Figure S4), stopped duration (Supplementary Figure S5) and duration of observation of study participants (Supplementary Figure S6).

**Table 3:** Meta-regression of people who restarted pre-exposure prophylaxis (N = 27).

Variable	Pooled proportion (95% CI)	Odds ratio for restarting PrEP (95% CI)	p	Adjusted odds ratio (95% CI)	p
Country income level					
High	0.26 (0.14, 0.40)	1	–	1	
Middle	0.17 (0.08, 0.28)	0.93 (0.80, 1.07)	0.294	**0**.**60 (0**.**50, 0**.**73)**	**<0**.**001**
World region					
Americas	0.25 (0.16, 0.35)	1		1	
Africa	0.25 (0.12, 0.41)	1.00 (0.85, 1.17)	0.999	**1**.**55 (1**.**30, 1**.**86)**	**<0**.**001**
Europe or Western Pacific	0.15 (0.13, 0.16)	0.89 (0.66, 1.19)	0.403	0.94 (0.79, 1.11)	0.429
Population type					
Mixed	0.25 (0.13, 0.38)	1		1	
MSM/TGW	0.27 (0.15, 0.40)	1.01 (0.84, 1.22)	0.901	1.11 (0.97, 1.27)	0.120
Heterosexual	0.30 (0.05, 0.65)	1.04 (0.80, 1.34)	0.770	**1**.**50 (1**.**25, 1**.**81)**	**<0**.**001**
AGYW	0.15 (0.05, 0.28)	0.89 (0.72, 1.09)	0.228	0.91 (0.78, 1.05)	0.174
Stopped duration					
≤1 month	0.31 (0.25, 0.39)	1		1	
>1 month	0.16 (0.10, 0.23)	0.85 (0.70, 1.04)	0.112	**1**.**20 (1**.**06, 1**.**36)**	**0**.**007**
Unclear	0.23 (0.12, 0.37)	0.95 (0.79, 1.13)	0.514	0.95 (0.83, 1.09)	0.416
Duration of observation of study participants					
<1 year	0.14 (0.08, 0.22)	1		–	
≥1 year	0.28 (0.19, 0.37)	1.17 (0.99, 1.37)	0.067	–	–
Unclear	0.20 (0.13, 0.29)	1.09 (0.76, 1.55)	0.629	–	–

MSM, men who have sex with men; TGW, transgender women; 95% CI, 95% confidence intervals.

Bolded text are variables that have a p-value of <0.05 in the multivariable model.

Supplementary Tables S1a and b summarise the quality assessment of studies. Briefly, the two randomised controlled trials had a low risk of bias. For the remaining 25 studies used in the meta-analysis, the most common potential risk of bias related to studies not explicitly addressing potential confounders (15/25, 60%) or not describing strategies to address incomplete follow-up (24/25, 96%).

### Reasons for restarting PrEP

Ten studies reported the reason for restarting PrEP.^15^^,^^22^^,^^36^^,^^38^, ^39^, ^40^, ^41^, ^42^, ^43^, ^44^ A major reason for restarting PrEP is related to perceived risk. For example, in a community study among young cisgender women in Kenya, a third of participants reported restarting PrEP when their partners returned home since they were unaware of their sexual partner's sexual behaviours while they were away from home.^42^ In a study from South Africa and Kenya, the main reasons for restarting PrEP included reconciliation with a past sexual partner, starting a new sexual relationship, restored access to PrEP services, and experiencing a heightened sense of HIV vulnerability, such as suspected partner infidelity or witnessing family or friend testing HIV positive.^36^ A study from the USA among gay and bisexual men noted that participants would consider retaking PrEP in the future if they decided to engage in higher–risk activities again, i.e., when they become sexually active or engage with multiple sexual partners.^41^ In a cohort analysis of young sexual and gender minorities assigned male at birth in the USA, the main predictors of restarting PrEP in the future included being in an open relationship or single, having access to health insurance, and having an HIV-positive partner.^22^ In a study involving heterosexual women in Kenya, restarting PrEP was associated with travelling in the past three months.^40^

The second major reason for restarting PrEP is related to the removal of barriers to PrEP access. In a study from New York, USA, eight (62%) participants restarted PrEP because their systemic issues had been resolved. The main systemic issues reported in the study included financial barriers and clinic/pharmacy access barriers.^39^ In a cross-sectional survey among GBM in Canada, a participant who restarted PrEP stated the reason as: ‘because I know things are getting better and we're heading soon to Stage 3 of reopening in Ontario’^43^ implying the elimination of lockdown related barriers was associated with restarting PrEP. In a cross-sectional survey in the USA, the reasons participants would consider restarting PrEP included accessing PrEP free of charge (33/55, 60%) and making PrEP available from the participant's primary care provider (7/55, 13%). Other reasons participants would consider restarting PrEP included if visits were less frequent (6/55, 11%), increased reminders from the clinic (6/55, 11%), providers encouraged PrEP use (5/55, 9%), and mental health/substance use counselling offered (3/55, 6%).^44^

Although we found no studies explicitly describing strategies to restart PrEP, a study from California, USA, described the impact of a survey sent to inactive PrEP clients. Approximately 25% of previous PrEP users restarted taking PrEP after completing the survey. The reasons why they restarted PrEP were not explored, but participating in the survey was reported as being associated with restarting PrEP.^39^

## Discussion

This systematic review identified 30 unique studies, with 27 reporting proportions of people restarting PrEP and 10 reporting reasons for restarting PrEP. We add to the scant literature on restarting PrEP by demonstrating an overall low proportion of restarting PrEP among people who stopped PrEP. Observed heterogeneity related to country-income level, world region, population type, and definition of restarting PrEP. We did not find any studies that evaluated strategies to support restarting PrEP. The main reasons for restarting PrEP are related to the perceived risk for HIV and the removal of structural barriers to access PrEP.

We estimated that the pooled proportion of restarting PrEP was 23.8% (95% CI: 15.9–32.7), which is lower than the 37% (95% CI: 22–52%) reported in an earlier systematic review by Zhang et al.,^9^ This could be because of the impact of the new studies. The earlier review included 8 studies, whereas this study identified 19 additional studies (totalling 27 studies), of which the studies not included in the previous review reported a lower proportion of people restarting PrEP (19.9%, 95% CI: 11.5–29.8, p = 0.057). We found high heterogeneity in our pooled estimate, mainly explained by the country's income level, world region, population type, and definition of someone who stopped PrEP. The socioeconomic and geographical variations in PrEP restarting reported in our study align with existing literature. First, individuals from middle-income countries had lower rates of restarting PrEP. This could be due to the lower rates of HIV in these countries.^45^ The higher restarting of PrEP in Africa compared to other regions could be due to the higher background HIV prevalence in this region, hence a need for individuals to take and stop PrEP more frequently as their perceived HIV risk may be higher. Conversely, this observation could be explained by relatively lower rates of restarting PrEP in populations from the USA, Europe, and Western Pacific due to poorer access to restarting PrEP among populations who remain at risk for HIV.

Heterosexuals were more likely to restart PrEP compared to MSM/TGW or studies with mixed populations (who were mainly MSM). We did not find any studies that explained why heterosexual couples may be more likely to restart PrEP than sexual minority groups. However, this observation could be due to the higher rates of PrEP discontinuation in heterosexuals. The relationship between sexual orientation, discontinuation and adherence to PrEP has been described in previous studies. In the study by Zhang et al., discontinuation rates were 72% among heterosexual individuals, compared to 31% among gay and bisexual men and 43% among women and adolescent girls.^9^ Similarly, since heterosexual individuals are less adherent to PrEP compared to MSM,^9^ this could lead to more frequent restarting in this population. Additionally, the higher rates of restarting in heterosexuals could indicate their more transient nature of HIV risk (compared to MSM/TGW) where the main reasons for restarting related to the return of a sexual partner from travel or a new partner. This finding could have policy and clinical implications for PrEP use. For example, while it is critical to use PrEP only when individuals are at perceived risk of HIV acquisition, efforts should be made to provide PrEP to the travelling partner during their time of travel away from usual sexual partners. Additionally, research should be undertaken on how best to support individuals, including accurate HIV risk assessment to inform appropriate and timely restarting of PrEP.

Understanding why people stop and restart PrEP is helpful to inform interventions to support PrEP users better. This is important, particularly if people are stopping PrEP for structural reasons while they remain at risk of HIV. We identified the most common reason for restarting PrEP and why individuals would consider restarting PrEP was a perceived higher risk of HIV acquisition. This has practical implications for PrEP programs to educate patients on how to stop and start PrEP in the context of accurate HIV risk assessment. Various HIV risk assessment tools could be used, for example, from the US Centres for Disease Control and Prevention.^46^ Further, online tools based on machine-learning algorithms are emerging to assist individuals in accurately assessing their own risk for HIV.^47^ Additionally, eliminating barriers to PrEP access was a common reason for individuals who restarted or would consider restarting PrEP in the future. This is an important finding to highlight, as prior studies have reported financing as a barrier to PrEP access.^48^ Further, people may choose not to restart PrEP because of infrequent risk exposures. Thus, options like Post Exposure Prophylaxis after Sexual Exposure (PEPSE)^49^ or PEP-in-pocket (PIP)^50^^,^^51^ may be preferred. Given the need to restart PrEP or the use of an alternate effective HIV prevention method whenever individual HIV risk exists or increases, it is important to document reasons why individuals restart PrEP and develop approaches to overcome any barriers where needed.

We did not find any studies that described strategies for re-starting PrEP. This highlights a significant gap within the literature. A few restarting strategies could be considered. First, web-based tools for real-time monitoring of PrEP users could support adherence and restarting for those who stop using PrEP.^52^ Using this platform can clearly document the pattern of PrEP use and rapidly identify people who may need to be prompted about their PrEP use. Second, telehealth could remove structural barriers to accessing PrEP and thus the ease of restarting PrEP.^53^ Telehealth enables overcoming geographic access barriers, increased scheduling flexibility, and reduced in-person contact—decreasing fear of discrimination.^53^ Third, encouraging the use of an HIV risk calculator allows individuals to educate themselves on their HIV risk so they can more accurately inform their decision-making on when to stop and start PrEP.^54^ Last, we need strategies aimed at addressing and removing barriers to access to PrEP usage, such as but not limited to addressing provider stigma, ensuring privacy and confidentiality, and lowering financial costs.

We conducted an additional literature search in March 2024 to update our literature review. We identified five additional studies.^55^, ^56^, ^57^, ^58^, ^59^ The findings of these studies did not change our conclusions. Four studies reported the proportion restarting, 0%,^56^ 8%,^55^ 18%^58^ and 21%,^59^ and one study from South Africa^57^ reported reasons for restarting PrEP. These reasons included increased familiarity with PrEP, encouragement from their social network, ability to manage side effects, self-initiated discussion with study staff, and shift in risk perception.

Our study should be read in light of some limitations. First, most studies were from two regions and do not reflect the current HIV epidemic, where there are rising HIV notifications in parts of Europe and the Western Pacific. More implementation science research from these regions is urgently needed to inform interventions to support restarting PrEP. Second, there was a lack of uniformity in defining restarting, with durations of stopping PrEP ranging from 14 to 90 days. Some people may have longer durations before needing to fill their PrEP script because of on-demand use or periodic use (e.g., only use when travelling). If not properly captured by each study, this may lead to an underestimation in our meta-analysis of those who restarted PrEP. Third, readers should be cautious about extrapolating our findings beyond the settings of included studies, with only 2 and 8 studies from LICs and MICs, respectively. We acknowledge the reasons for restarting PrEP are diverse and could vary across subpopulations' values and preferences, ease of accessing PrEP programs and associated costs, and other individual drivers of choice. There are also likely impacts based on variations in healthcare systems between countries. For example, most studies from HICs included in our review are from the USA. However, the USA's healthcare system differs from most HICs (i.e., it has less publicly funded services). Fourth, there was significant heterogeneity in the meta-analysis. Although our meta-regression identified factors that could partly explain this heterogeneity, there could be a risk of over-fitting in our model, indicating the need for future studies to verify our findings. Given the nature of studies included, other unmeasured confounders may explain the high heterogeneity for restarting PrEP (e.g., insurance status of PrEP users, perceived and actual stigma). Fifth, we used funnel plots and Egger's test to assess publication bias, commonly used in the published literature. However, there are arguments that funnel plots may not be the best approach for assessing publication bias in meta-analysis of proportions.^60^ Other tests like Doi plots and predictive intervals may offer a better alternative assessment.^60^^,^^61^ Sixth, the studies do not document the outcomes of those who restarted compared to those who did not restart PrEP. However, there is evidence that HIV incidence is higher among those who had stopped and did not restart PrEP.^25^^,^^31^^,^^62^ Future PrEP studies should clearly document patterns of PrEP use and if they employed strategies to follow up with those who stopped PrEP (e.g., determine ongoing risk for HIV and support for restarting PrEP where needed).

In conclusion, one in four PrEP users restarted PrEP after discontinuation, but significant differences were observed among countries and populations. Optimising the effectiveness of PrEP programs demands a better understanding of the underlying reasons and contextual factors that influence the decision to stop PrEP and establish effective support systems to support ongoing access to PrEP where there is continued need.

## Contributors

JJO conceived the idea. RK and QRS did the screening, data extraction and wrote the first draft of the manuscript. JJO conducted the statistical analysis. LZ accessed the analysed data and verified the analyses. All authors contributed to interpreting the results and subsequent edits of the manuscript and had final responsibility for the decision to submit for publication.

## Data sharing statement

Data will be made available upon request made to the corresponding author.

## Declaration of interests

The authors declare no conflicts of interest.
