# Supplementary material for: Restarting pre-exposure prophylaxis (PrEP) for HIV: a systematic review and meta-analysis
Source: eClinicalMedicine. 2024 May 17;72:102647. doi: 10.1016/j.eclinm.2024.102647 (PMC11127197; doi:10.1016/j.eclinm.2024.102647)
Supplement: Supplementary File [file mmc1.docx]

**Table of Contents**

[APPENDIX 1 1](#_Toc165366391)

[Supplementary Figure 1: Funnel plot of meta-analysis of 26 included studies 6](#_Toc165366392)

[Supplementary Figure 2: Subgroup analysis (country income) 7](#_Toc165366394)

[Supplementary Figure 3 Subgroup analysis (world region) 8](#_Toc165366395)

[Supplementary Figure 4 (population type) 9](#_Toc165366396)

[Supplementary Figure 5 (stopped duration) 10](#_Toc165366397)

[Supplementary Figure 6 Subgroup analysis (duration of observation of study participants) 11](#_Toc165366398)

[Supplementary Table 1a: Experimental / Randomized Controlled Trials 12](#_Toc165366399)

[Supplementary Table 1b: Cohort Studies 13](#_Toc165366400)

[Supplementary Table 2: Supplementary studies identified 16](#_Toc165366401)

[REFERENCES 17](#_Toc165366402)

## APPENDIX 1

**Ovid/EMBASE Search strategy– 13^th^ April, 2023**

1. hiv/ or hiv-1/ or hiv-2/
 2. hiv infections/ or acquired immunodeficiency syndrome/
 3. (human immunodeficiency virus* or human immunedeficiency virus* or human immuno-deficiency virus* or human immune-deficiency virus*).mp.
 4. (Acquired immunodeficiency syndrome* or acquired immunedeficiency syndrome* or acquired immuno-deficiency syndrome* or acquired immune-deficiency syndrome*).mp.
 5. (hiv or hiv-1 or hiv-2 or hiv1 or hiv2).mp.
 6. Hiv?[aids.mp](http://aids.mp/).
 7. 1 or 2 or 3 or 4 or 5 or 6
 8. Pre-Exposure Prophylaxis/
 9. (prophyla* adj5 (pre-exposure or pre?exposure)).mp.
 10. PREP.mp.
 11. 8 or 9 or 10
 12. (restart* or recommence* or re-establish* or reinstate* or re?establish* or re-instate* or re-commence* or re-initiat* or reinitiat* or re-start* or resum* or "begin again" or "start* again" or renew*).mp.
 13. 11 and 12
 14. anti-retroviral agents/ or anti-hiv agents/ or cobicistat/ or delavirdine/ or didanosine/ or efavirenz, emtricitabine, tenofovir disoproxil fumarate drug combination/ or elvitegravir, cobicistat, emtricitabine, tenofovir disoproxil fumarate drug combination/ or emtricitabine/ or emtricitabine, rilpivirine, tenofovir drug combination/ or emtricitabine, tenofovir disoproxil fumarate drug combination/ or lamivudine/ or nevirapine/ or raltegravir potassium/ or rilpivirine/ or stavudine/ or tenofovir/ or trichosanthin/ or zalcitabine/ or zidovudine/ or Truvada/
 15. 11 and 12 and 14
 16. ((pre-exposure or pre?exposure) and (anti-hiv or antihiv or cobicistat or delavirdine or didanosine or efavirenz or emtricitabine or tenofovir or disoproxil or elvitegravir or cobicistat or rilpivirine or lamivudine or nevirapine or raltegravir or stavudine or trichosanthin or zalcitabine or zidovudine)).mp.
 17. 12 and 16
 18. ((episodic* or intermittent*) adj4 ("use*" or "us?age" or "used" or "using" or utili?e* or utili?ation or utili?ing)).mp.
 19. 11 and 18
 20. (stop* adj2 start*).mp.
 21. 11 and 20
 22. 14 and 18
 23. 16 and 18
 24. 16 and 20
 25. 13 or 15 or 17 or 19 or 21 or 22 or 23 or 24

**282** results

**EBSCO/CINAHL Search strategy – 11^th^ April, 2023**

S1 hiv/ or hiv-1/ or hiv-2/

S2 hiv infections/ or acquired immunodeficiency syndrome/

S3 (human immunodeficiency virus* or human immunedeficiency virus* or human immuno-deficiency virus* or human immune-deficiency virus*)

S4 (Acquired immunodeficiency syndrome* or acquired immunedeficiency syndrome* or acquired immuno-deficiency syndrome* or acquired immune-deficiency syndrome*)

S5 (hiv or hiv-1 or hiv-2 or hiv1 or hiv2)

S6 hiv/aids

S7 S1 OR S2 OR S3 OR S4 OR S5 OR S6

S8 pre-exposure prophylaxis

S9 (prophyla* adj5 (pre-exposure or pre?exposure))

S10 PREP

S11 S8 OR S9 OR S10

S12 (restart* or recommence* or re-establish* or reinstate* or re?establish* or re-instate* or re-commence* or re-initiat* or reinitiat* or re-start* or resum* or "begin again" or "start* again" or renew*)

S13 S11 AND S12

S14 anti-retroviral agents/ or anti-hiv agents/ or cobicistat/ or delavirdine/ or didanosine/ or efavirenz, emtricitabine, tenofovir disoproxil fumarate drug combination/ or elvitegravir, cobicistat, emtricitabine, tenofovir disoproxil fumarate drug combination/ or emtricitabine/ or emtricitabine, rilpivirine, tenofovir drug combination/ or emtricitabine, tenofovir disoproxil fumarate drug combination/ or lamivudine/ or nevirapine/ or raltegravir potassium/ or rilpivirine/ or stavudine/ or tenofovir/ or trichosanthin/ or zalcitabine/ or zidovudine/ or Truvada/

S15 S11 AND S12 AND S14

S16 ((pre-exposure or pre?exposure) and (anti-hiv or antihiv or cobicistat or delavirdine or didanosine or efavirenz or emtricitabine or tenofovir or disoproxil or elvitegravir or cobicistat or rilpivirine or lamivudine or nevirapine or raltegravir or stavudine or trichosanthin or zalcitabine or zidovudine))

S17 S12 AND S16

S18 ((episodic* or intermittent*) adj4 ("use*" or "us?age" or "used" or "using" or utili?e* or utili?ation or utili?ing))

S19 S11 AND S18

S20 (stop* adj2 start*)

S21 S11 AND S20

S22 S14 AND S18

S23 S16 AND S18

S24 S16 AND S20

S25 S13 OR S15 OR S17 OR S19 OR S21 OR S22 OR S23 OR S24

**136** results

**Ovid/Emcare Search strategy– 13^th^ April, 2023**

1. hiv/ or hiv-1/ or hiv-2/
 2. hiv infections/ or acquired immunodeficiency syndrome/
 3. (human immunodeficiency virus* or human immunedeficiency virus* or human immuno-deficiency virus* or human immune-deficiency virus*).mp.
 4. (Acquired immunodeficiency syndrome* or acquired immunedeficiency syndrome* or acquired immuno-deficiency syndrome* or acquired immune-deficiency syndrome*).mp.
 5. (hiv or hiv-1 or hiv-2 or hiv1 or hiv2).mp.
 6. Hiv?[aids.mp](http://aids.mp/).
 7. 1 or 2 or 3 or 4 or 5 or 6
 8. Pre-Exposure Prophylaxis/
 9. (prophyla* adj5 (pre-exposure or pre?exposure)).mp.
 10. PREP.mp.
 11. 8 or 9 or 10
 12. (restart* or recommence* or re-establish* or reinstate* or re?establish* or re-instate* or re-commence* or re-initiat* or reinitiat* or re-start* or resum* or "begin again" or "start* again" or renew*).mp.
 13. 11 and 12
 14. anti-retroviral agents/ or anti-hiv agents/ or cobicistat/ or delavirdine/ or didanosine/ or efavirenz, emtricitabine, tenofovir disoproxil fumarate drug combination/ or elvitegravir, cobicistat, emtricitabine, tenofovir disoproxil fumarate drug combination/ or emtricitabine/ or emtricitabine, rilpivirine, tenofovir drug combination/ or emtricitabine, tenofovir disoproxil fumarate drug combination/ or lamivudine/ or nevirapine/ or raltegravir potassium/ or rilpivirine/ or stavudine/ or tenofovir/ or trichosanthin/ or zalcitabine/ or zidovudine/ or Truvada/
 15. 11 and 12 and 14
 16. ((pre-exposure or pre?exposure) and (anti-hiv or antihiv or cobicistat or delavirdine or didanosine or efavirenz or emtricitabine or tenofovir or disoproxil or elvitegravir or cobicistat or rilpivirine or lamivudine or nevirapine or raltegravir or stavudine or trichosanthin or zalcitabine or zidovudine)).mp.
 17. 12 and 16
 18. ((episodic* or intermittent*) adj4 ("use*" or "us?age" or "used" or "using" or utili?e* or utili?ation or utili?ing)).mp.
 19. 11 and 18
 20. (stop* adj2 start*).mp.
 21. 11 and 20
 22. 14 and 18
 23. 16 and 18
 24. 16 and 20
 25. 13 or 15 or 17 or 19 or 21 or 22 or 23 or 24

**80** results

**Elsevier/Scopus Search strategy– 11^th^ April, 2023**

TITLE-ABS-KEY ( ( restart* OR recommence* OR re-establish* OR reinstate* OR re-instate* OR re-commence* OR reinitiat* OR re-start* OR resum* OR renew* OR re-initiat* OR intermittent* OR episodic* ) AND ( pre exposure AND prophylaxis OR prophyla* OR prep OR truvada* OR emtricitabine OR tenofovir OR antihiv OR anti-hiv OR elvitegravir OR cobicistat OR reuse* OR re-use* ) )

**190** results

**Ovid/PhschINFO Search strategy– 13^th^ April, 2023**

1. hiv/ or hiv-1/ or hiv-2/
 2. hiv infections/ or acquired immunodeficiency syndrome/
 3. (human immunodeficiency virus* or human immunedeficiency virus* or human immuno-deficiency virus* or human immune-deficiency virus*).mp.
 4. (Acquired immunodeficiency syndrome* or acquired immunedeficiency syndrome* or acquired immuno-deficiency syndrome* or acquired immune-deficiency syndrome*).mp.
 5. (hiv or hiv-1 or hiv-2 or hiv1 or hiv2).mp.
 6. Hiv?[aids.mp](http://aids.mp/).
 7. 1 or 2 or 3 or 4 or 5 or 6
 8. Pre-Exposure Prophylaxis/
 9. (prophyla* adj5 (pre-exposure or pre?exposure)).mp.
 10. PREP.mp.
 11. 8 or 9 or 10
 12. (restart* or recommence* or re-establish* or reinstate* or re?establish* or re-instate* or re-commence* or re-initiat* or reinitiat* or re-start* or resum* or "begin again" or "start* again" or renew*).mp.
 13. 11 and 12
 14. anti-retroviral agents/ or anti-hiv agents/ or cobicistat/ or delavirdine/ or didanosine/ or efavirenz, emtricitabine, tenofovir disoproxil fumarate drug combination/ or elvitegravir, cobicistat, emtricitabine, tenofovir disoproxil fumarate drug combination/ or emtricitabine/ or emtricitabine, rilpivirine, tenofovir drug combination/ or emtricitabine, tenofovir disoproxil fumarate drug combination/ or lamivudine/ or nevirapine/ or raltegravir potassium/ or rilpivirine/ or stavudine/ or tenofovir/ or trichosanthin/ or zalcitabine/ or zidovudine/ or Truvada/
 15. 11 and 12 and 14
 16. ((pre-exposure or pre?exposure) and (anti-hiv or antihiv or cobicistat or delavirdine or didanosine or efavirenz or emtricitabine or tenofovir or disoproxil or elvitegravir or cobicistat or rilpivirine or lamivudine or nevirapine or raltegravir or stavudine or trichosanthin or zalcitabine or zidovudine)).mp.
 17. 12 and 16
 18. ((episodic* or intermittent*) adj4 ("use*" or "us?age" or "used" or "using" or utili?e* or utili?ation or utili?ing)).mp.
 19. 11 and 18
 20. (stop* adj2 start*).mp.
 21. 11 and 20
 22. 14 and 18
 23. 16 and 18
 24. 16 and 20
 25. 13 or 15 or 17 or 19 or 21 or 22 or 23 or 24

**31** results

**Ovid/MEDLINE Search strategy– 6^th^ April, 2023**

1. hiv/ or hiv-1/ or hiv-2/
 2. hiv infections/ or acquired immunodeficiency syndrome/
 3. (human immunodeficiency virus* or human immunedeficiency virus* or human immuno-deficiency virus* or human immune-deficiency virus*).mp.
 4. (Acquired immunodeficiency syndrome* or acquired immunedeficiency syndrome* or acquired immuno-deficiency syndrome* or acquired immune-deficiency syndrome*).mp.
 5. (hiv or hiv-1 or hiv-2 or hiv1 or hiv2).mp.
 6. Hiv?[aids.mp](http://aids.mp/).
 7. 1 or 2 or 3 or 4 or 5 or 6
 8. Pre-Exposure Prophylaxis/
 9. (prophyla* adj5 (pre-exposure or pre?exposure)).mp.
 10. PREP.mp.
 11. 8 or 9 or 10
 12. (restart* or recommence* or re-establish* or reinstate* or re?establish* or re-instate* or re-commence* or re-initiat* or reinitiat* or re-start* or resum* or "begin again" or "start* again" or renew*).mp.
 13. 11 and 12
 14. anti-retroviral agents/ or anti-hiv agents/ or cobicistat/ or delavirdine/ or didanosine/ or efavirenz, emtricitabine, tenofovir disoproxil fumarate drug combination/ or elvitegravir, cobicistat, emtricitabine, tenofovir disoproxil fumarate drug combination/ or emtricitabine/ or emtricitabine, rilpivirine, tenofovir drug combination/ or emtricitabine, tenofovir disoproxil fumarate drug combination/ or lamivudine/ or nevirapine/ or raltegravir potassium/ or rilpivirine/ or stavudine/ or tenofovir/ or trichosanthin/ or zalcitabine/ or zidovudine/ or Truvada/
 15. 11 and 12 and 14
 16. ((pre-exposure or pre?exposure) and (anti-hiv or antihiv or cobicistat or delavirdine or didanosine or efavirenz or emtricitabine or tenofovir or disoproxil or elvitegravir or cobicistat or rilpivirine or lamivudine or nevirapine or raltegravir or stavudine or trichosanthin or zalcitabine or zidovudine)).mp.
 17. 12 and 16
 18. ((episodic* or intermittent*) adj4 ("use*" or "us?age" or "used" or "using" or utili?e* or utili?ation or utili?ing)).mp.
 19. 11 and 18
 20. (stop* adj2 start*).mp.
 21. 11 and 20
 22. 14 and 18
 23. 16 and 18
 24. 16 and 20
 25. 13 or 15 or 17 or 19 or 21 or 22 or 23 or 24

**199** results

**Grey literature search**

Sources of grey literature were searched, including the AIDS conference and ID week meeting proceedings. We searched conference proceedings from 2010 to 2023. The search words used for searching conference books and websites were “PrEP” and “restarting”. From this, we identified 9 related abstracts. After assessing for duplication, we found that all 9 abstracts had been previously identified in our database.

We also searched Google Scholar and reviewed the first 200 items for any new articles. We used the following key words for the Google Scholar Search (HIV, PrEP, restating). We did not identify additional items after reviewing the first 200 items. We searched the WHO website (<https://www.who.int/data/gho/data/themes/hiv-aids/data-on-the-hiv-aids-response>) and UNAIDS website (<https://www.unaids.org/en/taxonomy/term/702>) for additional grey literature. No new records were identified during this search either.

## Supplementary Figure 1: Funnel plot of meta-analysis of 26 included studies

##
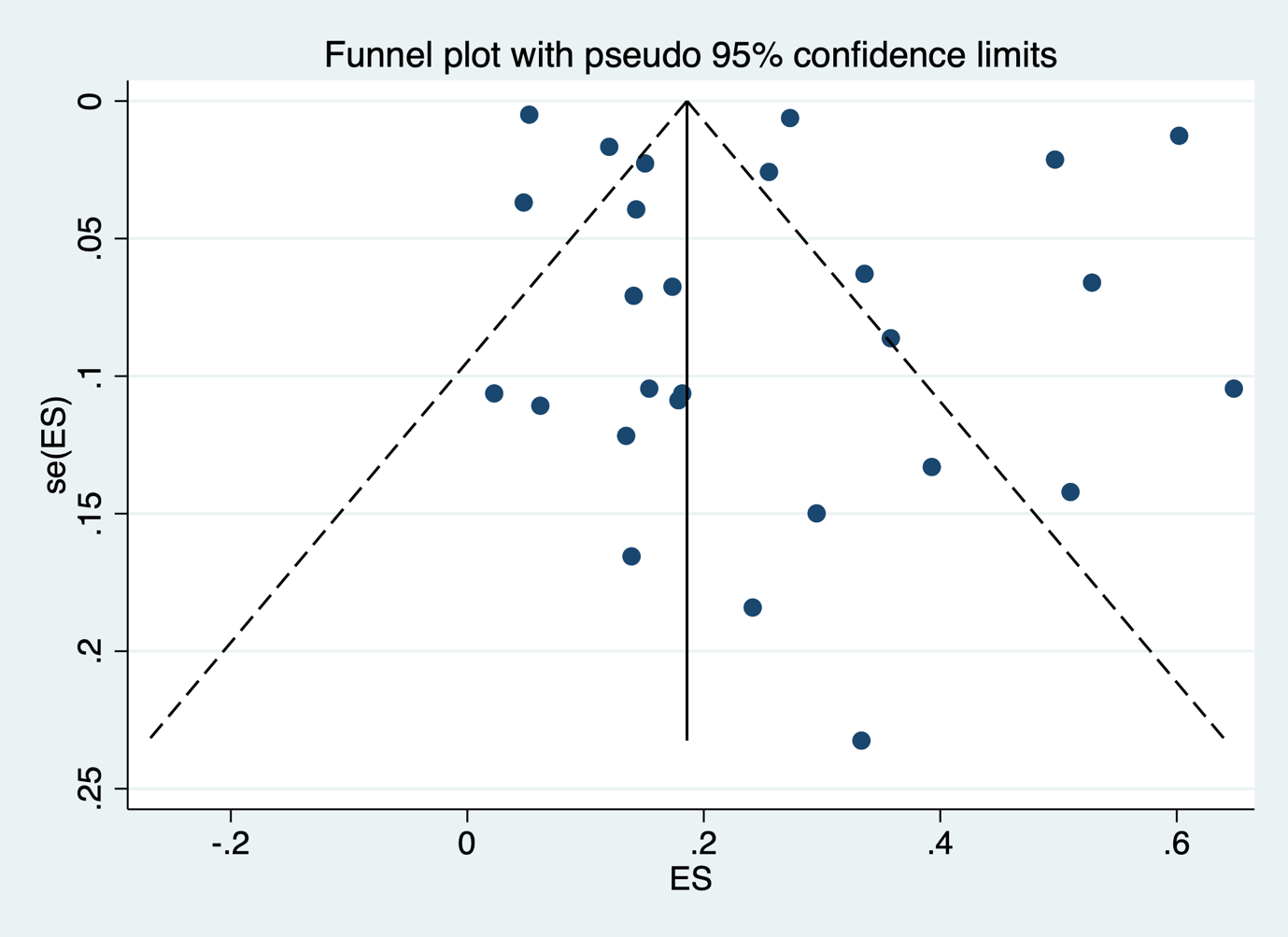


Egger’s test , p=0.51

## Supplementary Figure 2: Subgroup analysis (country income)

**
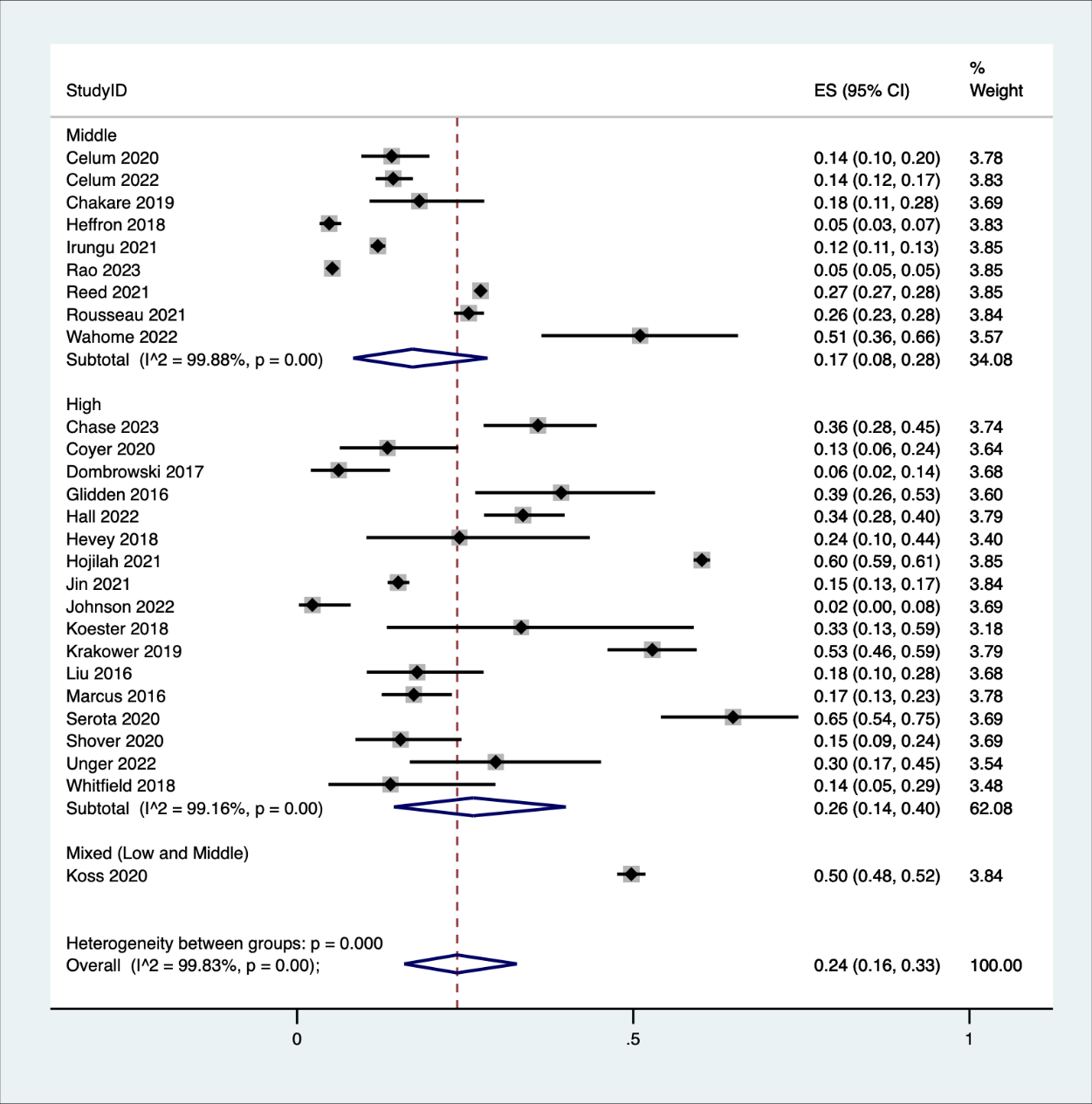
**

## Supplementary Figure 3 Subgroup analysis (world region)

**
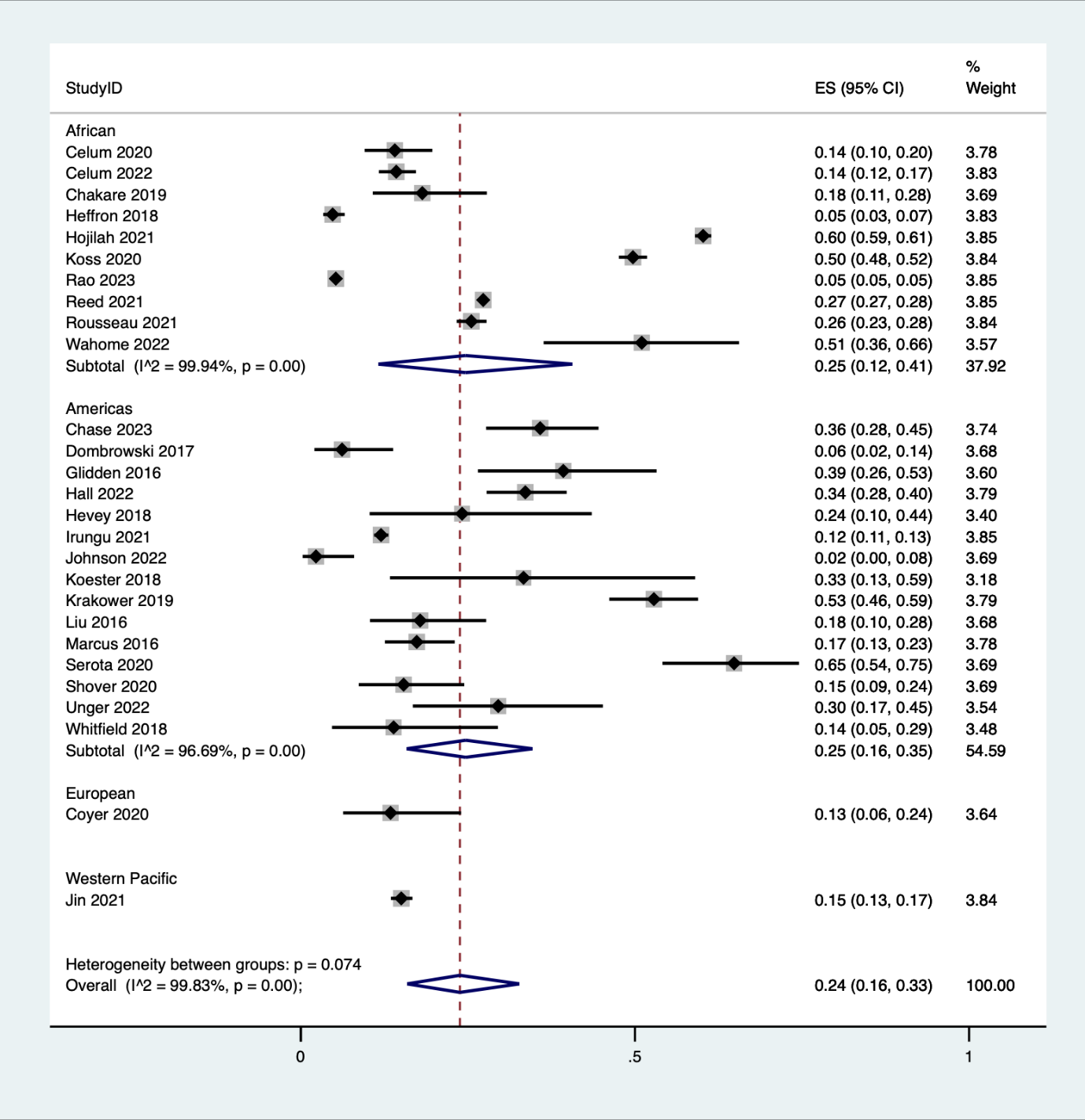
**

## Supplementary Figure 4 (population type)


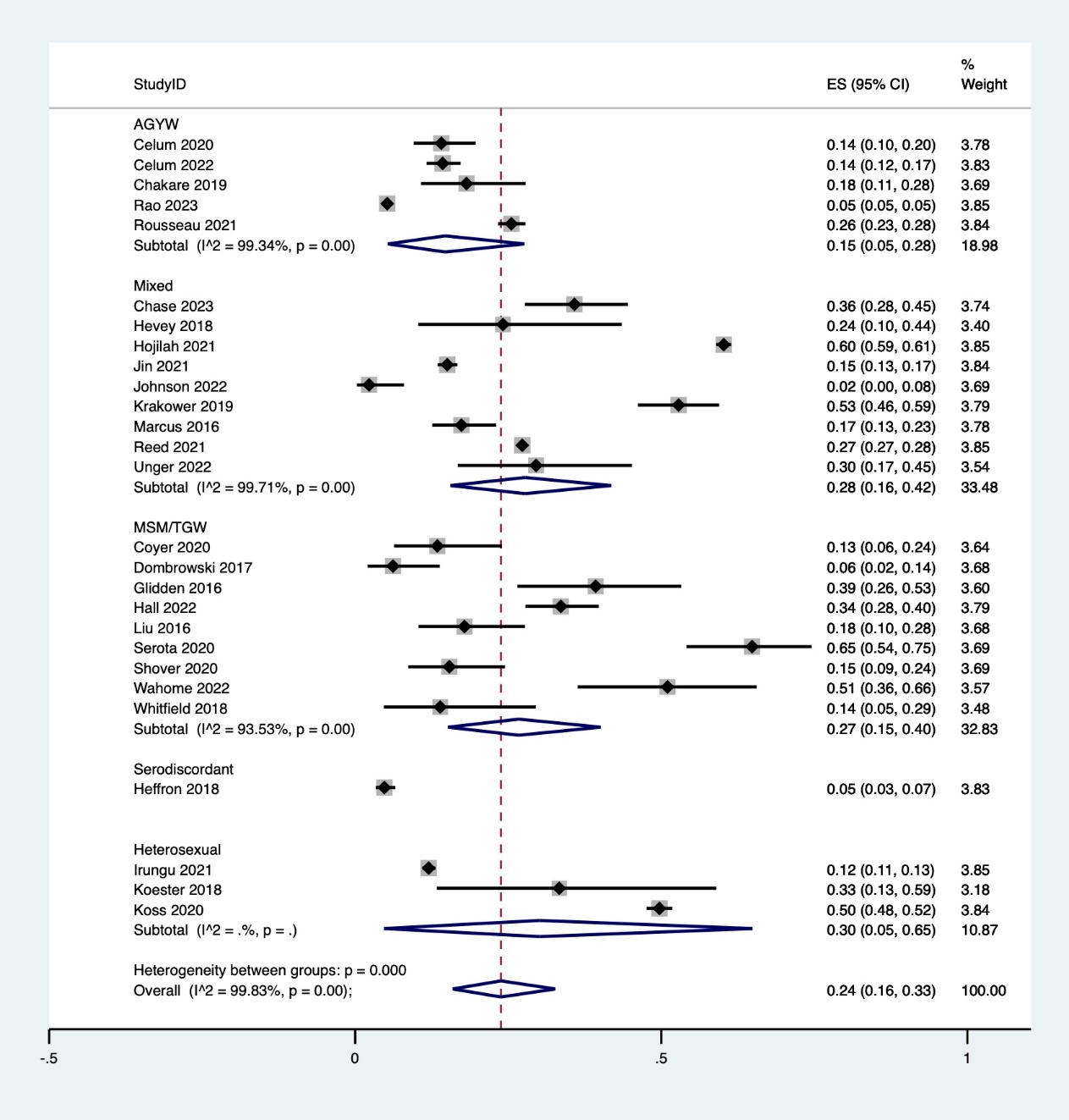


AGYW = adolescent girls and young women; MSM = men who have sex with men; TGW = transgender women

## Supplementary Figure 5 (stopped duration)

**
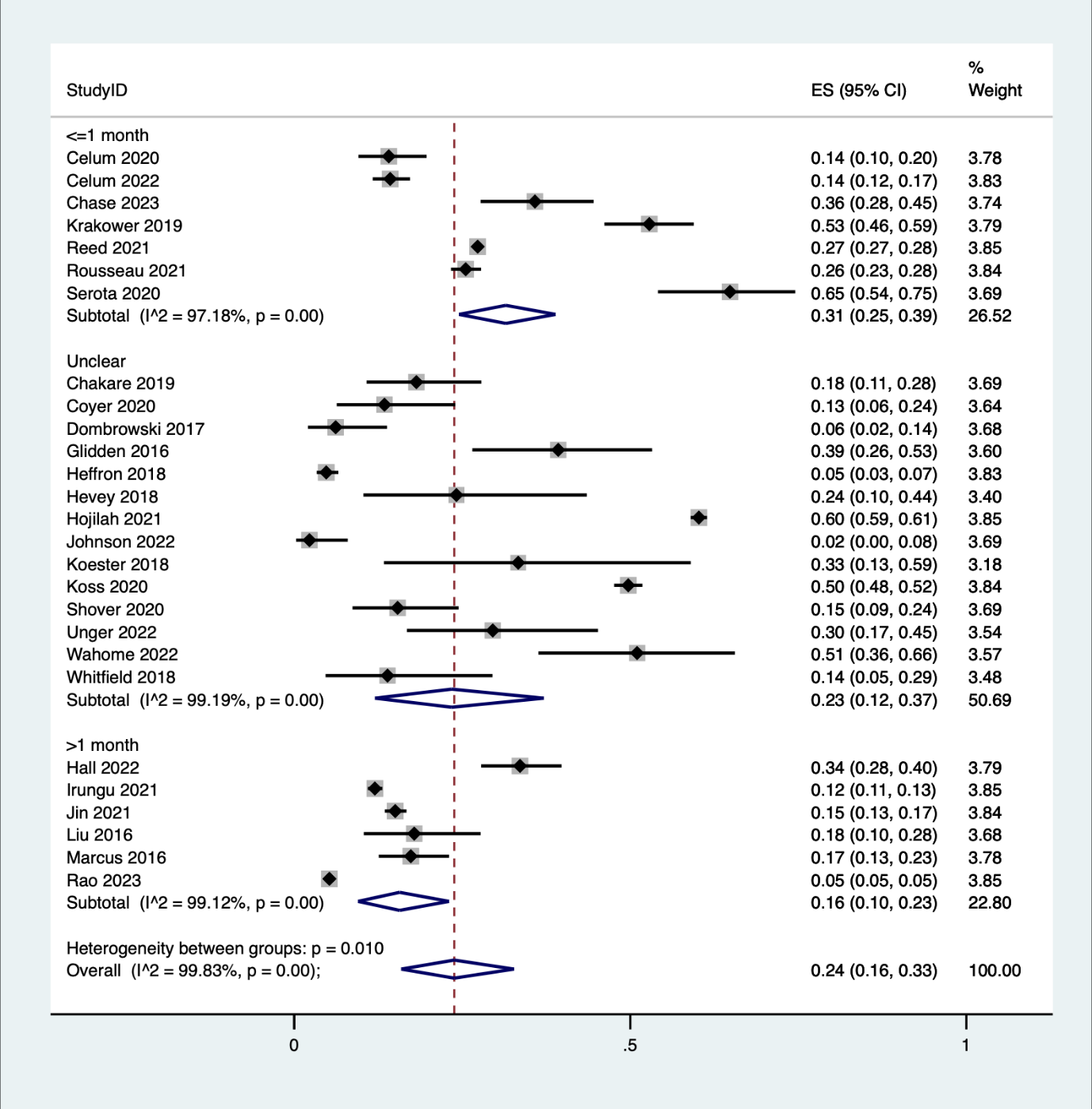
**

## Supplementary Figure 6 Subgroup analysis (duration of observation of study participants)

## **
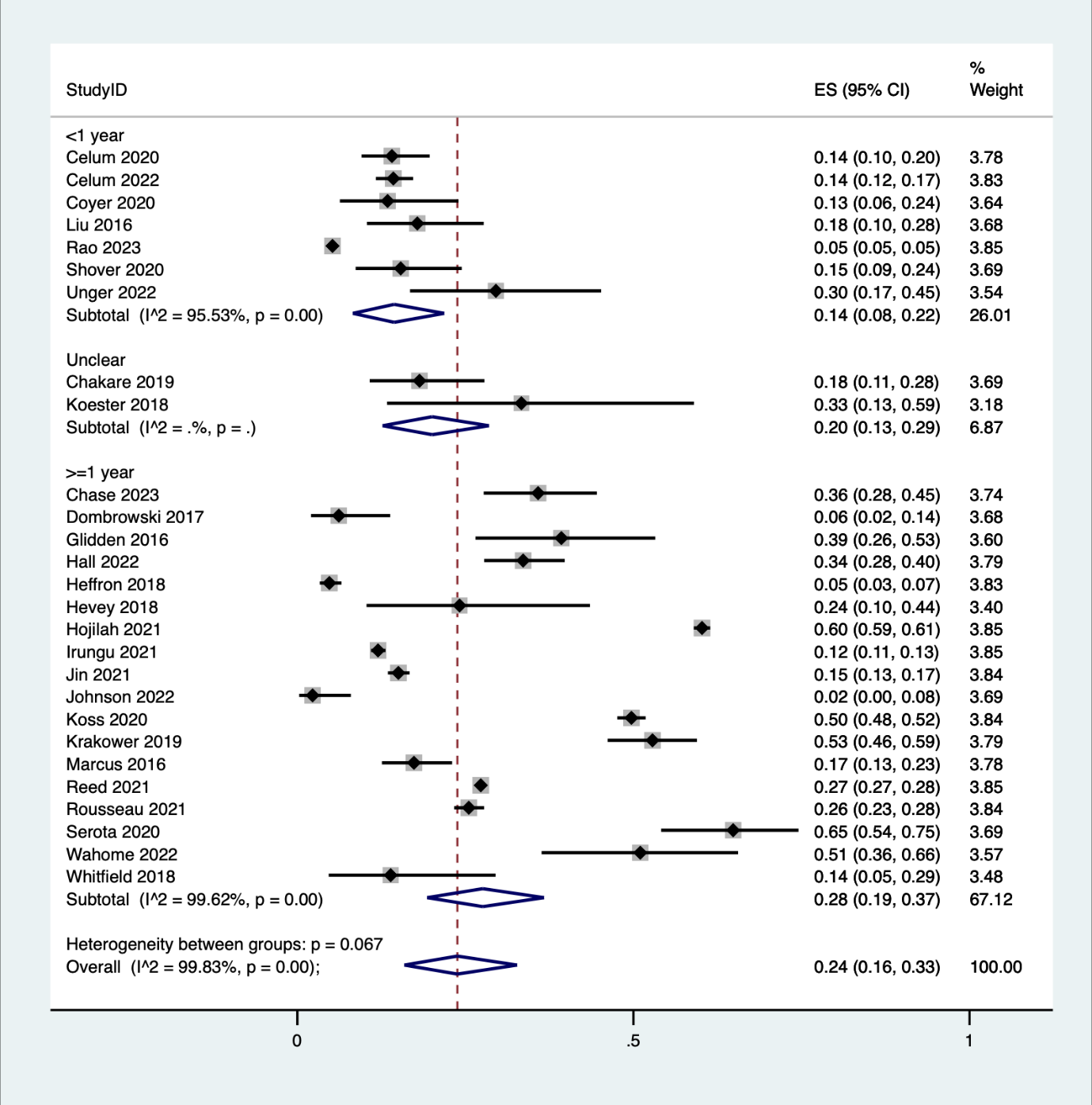
**

## Supplementary Table 1a: Experimental / Randomized Controlled Trials

| Author (et al.) | Year of Publication | Title | Study Type | Q1 | Q2 | Q3 | Q4 | Q5 | Q6 | Q7 | Q8 | Q9 | Q10 | Q11 | Q12 | Q13 |
| --- | --- | --- | --- | --- | --- | --- | --- | --- | --- | --- | --- | --- | --- | --- | --- | --- |
| *Irungu* | *2021* | *Integration of pre-exposure prophylaxis services into public HIV care clinics in Kenya: a pragmatic stepped-wedge randomised trial* | *1* | Y | N | Y | N | N | Y | U | Y | Y | Y | Y | Y | Y |
| *Celum* | *2020* | *Incentives conditioned on tenofovir levels to support PrEP adherence among young South African women: a randomized trial* | *1* | Y | Y | Y | Y | U | Y | U | Y | Y | Y | Y | Y | Y |

Key

- Study type:
  - 1= Exp/RCT - Control or Comparator Group included.
    2= Non-experimental/Cross-sectional - no control/comparator group.
    3= Modelling
    4= Qualitative
    5= Others
- Q1: Was true randomization used for assignment of participants to treatment groups?
- Q2: Was allocation to treatment groups concealed?
- Q3: Were treatment groups similar at the baseline?
- Q4: Were participants blind to treatment assignment?
- Q5: Were those delivering the treatment blind to treatment assignment?
- Q6: Were treatment groups treated identically other than the intervention of interest?
- Q7: Were outcome assessors blind to treatment assignment?
- Q8: Were outcomes measured in the same way for treatment groups?
- Q9: Were outcomes measured in a reliable way?
- Q10: Was follow up complete and if not, were differences between groups in terms of their follow up adequately described and analysed?
- Q11: Were participants analysed in the groups to which they were randomized?
- Q12: Was appropriate statistical analysis used?
- Q13: Was the trial design appropriate and any deviations from the standard RCT design (individual randomization, parallel groups) accounted for in the conduct and analysis of the trial?
- Y = Yes
- N = No
- U = Unclear
- NA = Not applicable

## Supplementary Table 1b: Cohort Studies

| Author | Year of Publication | Title | Study type | Q1 | Q2 | Q3 | Q4 | Q5 | Q6 | Q7 | Q8 | Q9 | Q10 | Q11 |
| --- | --- | --- | --- | --- | --- | --- | --- | --- | --- | --- | --- | --- | --- | --- |
| Celum | 2022 | *Prep use and HIV seroconversion rates in adolescent girls and young women from Kenya and South Africa: the power demonstration project* | 2 | Y | Y | Y | Y | U | Y | Y | Y | U | U | Y |
| Chase | 2023 | *Patterns of Pre-exposure Prophylaxis (PrEP) Use in a Population Accessing PrEP in Jackson, Mississippi* | 2 | Y | Y | Y | U | U | Y | Y | Y | U | U | Y |
| Dombrowski | 2017 | *Loss to follow-up and patient self-discontinuation of HIV pre-exposure prophylaxis (PREP) in an std clinic-based prep program with adherence support.* | 2 | Y | Y | Y | U | U | Y | Y | Y | U | U | Y |
| Hojilah | 2021 | *Characterization of HIV Preexposure Prophylaxis Use Behaviors and HIV Incidence Among US Adults in an Integrated Health Care System* | 2 | Y | Y | Y | Y | Y | Y | Y | Y | U | U | Y |
| Jin | 2021 | *Adherence to daily HIV pre-exposure prophylaxis in a large-scale implementation study in New South Wales, Australia* | 2 | Y | Y | Y | Y | Y | Y | Y | Y | U | U | Y |
| Johnson | 2022 | *Texting lost-to-follow-up PrEP patients from a San Francisco sexual health clinic* | 2 | Y | Y | Y | Y | Y | Y | Y | Y | Y | U | Y |
| Koester | 2018 | *Use of primary care providers to promote access to HIV pre-exposure prophylaxis (PrEP) among female patients in Oakland, California, United States* | 2 | Y | Y | U | U | U | U | U | Y | U | U | Y |
| Rao | 2023 | *Longitudinal patterns of initiation, persistence, and cycling on preexposure prophylaxis among female sex workers and adolescent girls and young women in South Africa.* | 2 | Y | Y | Y | U | U | Y | Y | Y | U | U | Y |
| Reed | 2021 | *HIV PrEP is more than ART-lite: Longitudinal study of real-world PrEP services data identifies missing measures meaningful to HIV prevention programming* | 2 | Y | Y | Y | U | U | Y | Y | Y | U | U | Y |
| Rousseau | 2021 | *Adolescent girls and young women's PrEP-user journey during an implementation science study in South Africa and Kenya.* | 2 | Y | Y | Y | N | U | Y | Y | Y | U | Y | Y |
| Shover | 2020 | *Structural, dosing, and risk change factors affecting discontinuation of pre-exposure prophylaxis (Prep) in a large urban clinic* | 2 | Y | Y | Y | U | U | Y | Y | Y | U | U | Y |
| Whitfield | 2018 | *Why I Quit Pre-Exposure Prophylaxis (PrEP)? A Mixed-Method Study Exploring Reasons for PrEP Discontinuation and Potential Re-initiation Among Gay and Bisexual Men* | 2 | Y | Y | Y | U | U | Y | Y | Y | U | U | Y |
| Unger | 2022 | *Reasons for PrEP Discontinuation After Navigation at Sexual Health Clinics: Interactions Among Systemic Barriers, Behavioral Relevance, and Medication Concerns* | 2 | Y | Y | Y | U | U | Y | Y | Y | N | U | Y |
| Wahome | 2022 | *Stopping and restarting PrEP and loss to follow-up among PrEP-taking men who have sex with men and transgender women at risk of HIV-1 participating in a prospective cohort study in Kenya* | 2 | Y | Y | Y | U | U | Y | Y | Y | U | U | Y |
| Hevey | 2018 | *PrEP continuation, HIV and STI testing rates, and delivery of preventive care in a clinic-based cohort* | 2 | Y | Y | Y | U | U | Y | Y | Y | U | U | Y |
| Marcus | 2016 | *Preexposure Prophylaxis for HIV Prevention in a Large Integrated Health Care System: Adherence, Renal Safety, and Discontinuation* | 2 | Y | Y | Y | U | U | Y | Y | Y | Y | U | Y |
| Glidden | 2016 | *Symptoms, Side Effects and Adherence in the iPrEx Open-Label Extension* | 2 | Y | Y | Y | U | U | Y | Y | Y | U | U | Y |
| Krakower | 2019 | *Patterns and clinical consequences of discontinuing HIV preexposure prophylaxis during primary care* | 2 | Y | Y | Y | U | U | Y | Y | Y | U | U | Y |
| Serota | 2020 | Pre-exposure Prophylaxis Uptake and Discontinuation Among Young Black Men Who Have Sex With Men in Atlanta, Georgia: A Prospective Cohort Study | 2 | Y | Y | Y | N | U | Y | U | Y | U | U | Y |
| Koss | 2020 | *Uptake, engagement, and adherence to pre-exposure prophylaxis offered after population HIV testing in rural Kenya and Uganda: 72-week interim analysis of observational data from the SEARCH study* | 2 | Y | Y | Y | Y | Y | Y | U | U | U | U | Y |
| Hall | 2022 | *Predictors of Re-Initiation of Daily Oral Preexposure Prophylaxis Regimen After Discontinuation* | 2 | Y | Y | Y | Y | Y | Y | Y | Y | U | U | Y |
| Heffron | 2018 | *Pre-exposure prophylaxis for HIV-negative persons with partners living with HIV: uptake, use, and effectiveness in an open-label demonstration project in East Africa* | 2 | Y | Y | Y | Y | U | Y | Y | Y | U | U | Y |
| Chakare | 2019 | *HIV risk perception and salience are paradoxically associated with Pre-Exposure Prophylaxis (PrEP) discontinuation among adolescent girls and young women in Lesotho* | 2 | Y | Y | Y | N | U | Y | U | U | U | U | Y |
| Liu | 2016 | *Preexposure Prophylaxis for HIV Infection Integrated With Municipal- and Community-Based Sexual Health Services* | 2 | Y | Y | Y | Y | Y | Y | Y | Y | U | U | Y |
| Coyer | 2020 | *Understanding pre-exposure prophylaxis (PrEP) regimen use: Switching and discontinuing daily and event-driven PrEP among men who have sex with men* | 2 | Y | Y | Y | U | U | Y | Y | Y | U | U | Y |

Key

- Study type:
  - 1= Exp/RCT - Control or Comparator Group included.
    2= Non-experimental/Cross-sectional - no control/comparator group.
    3= Modelling
    4= Qualitative
    5= Others
- Q1: Were the two groups similar and recruited from the same population?
- Q2: Were the exposures measured similarly to assign people to both exposed and unexposed groups?
- Q3: Was the exposure measured in a valid and reliable way?
- Q4: Were confounding factors identified?
- Q5: Were strategies to deal with confounding factors stated?
- Q6: Were the groups/participants free of the outcome at the start of the study (or at the moment of exposure)?
- Q7: Were the outcomes measured in a valid and reliable way?
- Q8: Was the follow up time reported and sufficient to be long enough for outcomes to occur?
- Q9: Was follow up complete, and if not, were the reasons to loss to follow up described and explored?
- Q10: Were strategies to address incomplete follow up utilized?
- Q11: Was appropriate statistical analysis used?
- Y = Yes
- N = No
- U = Unclear
- NA = Not applicable

## Supplementary Table 2: Supplementary studies identified

| **Author(Year)** | **Country** | **Enrollment** | **Sample size** | **Study type** | **Duration of follow-up** | **Population** | **Definition of restarting** | **Proportion of population** | **Reasons for restarting** |
| --- | --- | --- | --- | --- | --- | --- | --- | --- | --- |
| Khadka, 2023^1^ | South Africa | August 2019 to October 2021 | 1200 | Prospective cohort | 12 months | Pregnant and postpartum adolescent girls and young women | Missing a study visit at either 1 or 3 months. | 8% stopped and restarted on PrEP use at 6 months | Not provided |
| Colver, 2023^2^ | USA | 2020-2022 | 112 | Prospective cohort | 24 months | Military service members | Not defined | 0% | Not provided |
| de Vos, 2023^3^ | South Africa | December 2019 and April 2021 | 50 | In-depth interviews of individuals enrolled in a prospective cohort | 17 months | AGYW (aged 16–25) enrolled in the Community PrEP Study | Not provided | Not provided | - Increased familiarity with PrEP   *e.g., additional information*   - Encourage by social network. - Ability to manage side effects. - Self-initiated discussion with study staff   *e.g., side effects, temporary movement*   - Shift in risk perception.   *e.g., high risk, relationship status, pregnancy* |
| Cox, 2023^4^ | South Africa | July 2020  and May 2022 | 364 | Prospective cohort | 22 months | Men | PrEP restart  was defined as a return visit and PrEP issue 31 days or more after  the scheduled pick-up | 18% | Not provided |
| Ortblad, 2023^5^ | Kenya | November 2020 to October 2021 | 287 | Community based prospective study | 11 months | Men and women | Refilling PrEP more than 15 days after a scheduled visit | 21% (61/287) | Not provided |

## REFERENCES

1. Khadka N, Gorbach PM, Nyemba DC, et al. Evaluating the use of oral pre-exposure prophylaxis among pregnant and postpartum adolescent girls and young women in Cape Town, South Africa. *Front Reprod Health* 2023; **Sep 19;5:1224474**.

2. Colver JT, Yabes J, Marcus J. Persistence with Human Immunodeficiency Virus Pre-Exposure Prophylaxis in an Active-Duty Military Population. Open Forum Infect Dis. 2023 **Nov 27;10**(Suppl 2):ofad500.1370. doi: 10.1093/ofid/ofad500.1370.

3. de Vos L, Mudzingwa EK, Fynn L, et al. Factors that influence adolescent girls and young women's re-initiation or complete discontinuation from daily oral PrEP use: a qualitative study from Eastern Cape Province, South Africa. *J Int AIDS Soc* 2023; (9): e26175.

4. Cox LA, Martin CE, Nongena P, Mvelase S, Kutywayo A, Mullick S. The Use of HIV Pre-exposure Prophylaxis Among Men Accessing Routine Sexual and Reproductive Health Services in South Africa. *J Adolesc Health* 2023; **Dec;73**(6S): S92-S100.

5. Ortblad KF, Mogere P, Omollo V, et al. Stand-alone model for delivery of oral HIV pre-exposure prophylaxis in Kenya: a single-arm, prospective pilot evaluation. *J Int AIDS Soc* 2023; **Aug;26**(8): e26169.
